# *S1P* defects cause a new entity of cataract, alopecia, oral mucosal disorder, and psoriasis‐like syndrome

**DOI:** 10.15252/emmm.202114904

**Published:** 2022-04-01

**Authors:** Fuying Chen, Cheng Ni, Xiaoxiao Wang, Ruhong Cheng, Chaolan Pan, Yumeng Wang, Jianying Liang, Jia Zhang, Jinke Cheng, Y Eugene Chin, Yi Zhou, Zhen Wang, Yiran Guo, She Chen, Stephanie Htun, Erin F Mathes, Alejandra G de Alba Campomanes, Anne M Slavotinek, Si Zhang, Ming Li, Zhirong Yao

**Affiliations:** ^1^ Department of Dermatology Xinhua Hospital Shanghai Jiaotong University School of Medicine Shanghai China; ^2^ Institute of Dermatology Shanghai Jiaotong University School of Medicine Shanghai China; ^3^ Shanghai Key Laboratory for Tumor Microenvironment and Inflammation Department of Biochemistry and Molecular Cell Biology Shanghai Jiao Tong University School of Medicine Shanghai China; ^4^ Instituteof Health Sciences, Chinese Academy of Sciences Shanghai Jiaotong University School of Medicine Shanghai China; ^5^ Department of gastroenterology Zhongshan Hospital Fudan University Shanghai China; ^6^ Department of Dermatology Children's Hospital of Shanghai Jiaotong University Shanghai China; ^7^ Center for Data Driven Discovery in Biomedicine Children’s Hospital of Philadelphia PA USA; ^8^ NHC Key Laboratory of Glycoconjugate Research Department of Biochemistry and Molecular Biology School of Basic Medical Sciences Fudan University Shanghai China; ^9^ Division of Genetics Department of Pediatrics University of California San Francisco San Francisco CA USA; ^10^ Departments of Dermatology and Pediatrics University California San Francisco CA USA; ^11^ Department of Ophthalmology University of California San Francisco CA USA

**Keywords:** CAOP, electron transfer flavoprotein, MBTPS1, mitochondrial respiratory chain reaction, Genetics, Gene Therapy & Genetic Disease, Organelles

## Abstract

In this report, we discovered a new entity named cataract, alopecia, oral mucosal disorder, and psoriasis‐like (CAOP) syndrome in two unrelated and ethnically diverse patients. Furthermore, patient 1 failed to respond to regular treatment. We found that CAOP syndrome was caused by an autosomal recessive defect in the mitochondrial membrane‐bound transcription factor peptidase/site‐1 protease (*MBTPS1*, S1P). Mitochondrial abnormalities were observed in patient 1 with CAOP syndrome. Furthermore, we found that S1P is a novel mitochondrial protein that forms a trimeric complex with ETFA/ETFB. S1P enhances ETFA/ETFB flavination and maintains its stability. Patient S1P variants destabilize ETFA/ETFB, impair mitochondrial respiration, decrease fatty acid β‐oxidation activity, and shift mitochondrial oxidative phosphorylation (OXPHOS) to glycolysis. Mitochondrial dysfunction and inflammatory lesions in patient 1 were significantly ameliorated by riboflavin supplementation, which restored the stability of ETFA/ETFB. Our study discovered that mutations in *MBTPS1* resulted in a new entity of CAOP syndrome and elucidated the mechanism of the mutations in the new disease.

## Graphical abstract

Autosomal recessive variants in MBTPS1 gene cause a new entity of cataract, alopecia, oral mucosal disorder and psoriasis‐like (CAOP) syndrome. Further in vitro and in vivo analyses confirmed the pathogenicity of MBTPS1 variants. Our study also demonstrates that the CAOP syndrome patient was responsive to riboflavin treatment.

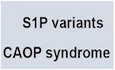

**The paper explained**

**Problem**

CAOP syndrome is characterized by ocular abnormalities, noncicatricial generalized alopecia, oral mucosal disorder, and psoriasiform skin lesions. Long‐term follow‐up is necessary for these patients, and controlled trials are needed with a broader range of patients to better understand the potential long‐term efficacy of riboflavin supplementation.

**Results**

In this report, we discovered a new entity named CAOP syndrome in a 13‐year‐old Chinese boy. Furthermore, the patient 1 failed to respond to the regular treatment. We found that the CAOP syndrome is caused by autosomal recessive defects in mitochondrial *MBTPS1*. MBTPS1 interacts with and activates ETFB. As a result, dysfunction of MBTPS1 in the patient disrupted the electron transport chain, leading to decreased ATP production, and increased reactive oxygen species. Consistent with these findings, abnormalities in lipid metabolism and in mitochondria were observed. Finally, the patient 1 was responsive to riboflavin treatment, which is a cofactor of ETFB.

**Impact**

To our knowledge, this is the first study to report *MBTPS1* as a disease‐causing gene of CAOP syndrome. We showed that MBTPS1 regulates lipid metabolism and mitochondrial function by activating ETFB. Our study is also the first to demonstrate that the *MBTPS*1 variant patient was responsive to riboflavin treatment, which is the cofactor of ETFB.

## Introduction

Two sporadic cases, a 14‐year‐old Chinese male patient (patient 1) and a 5‐year‐old Hispanic female patient (patient 2), were first diagnosed with cataract, alopecia, oral mucosal disorder, and psoriasis‐like (CAOP) syndrome, which was characterized by early‐onset bilateral lens cataract, generalized nonscarring alopecia, oral mucosal disorder, and severe psoriasiform skin lesions affecting the scalp, facial, inguinal region, buttocks, and lower extremities. Hematoxylin–eosin (HE)‐stained sections of the skin of patient 1 showed psoriasiform perivasculitis. To the best of our knowledge, the patients’ clinical symptoms were different from those of all the known dermatoses, and patient 1 was unresponsive to regular treatment, including oral acitretin, zinc sulfate, and topical steroids. Whole‐exome sequencing and Sanger sequencing of both patients identified compound heterozygous variants in the membrane‐embedded zinc metalloprotease/site‐1 protease (*MBTPS1/*S1P) gene in these patients.

S1P is essential for the regulation of cholesterol homeostasis and endoplasmic reticulum (ER) stress responses. S1P catalyzes the first step in the proteolytic activation of transcription factor sterol regulatory element‐binding proteins (SREBPs) and the first step in the proteolytic activation of cyclic AMP‐dependent activating transcription factor 6 (ATF6), which is confirmed by the variants identified in patients 1 and 2. S1P has also been proven to be a regulator of lysosome biogenesis via proteolytic activation of the hexametric GlcNAc‐1‐phosphotransferase complex, which is needed for the modification of newly synthesized lysosomal enzymes (Yang *et al*, 2001; Marschner *et al*, 2011). The role of S1P in mitochondrial function has not yet been reported.

The mitochondrial electron transport chain is essential for ATP production and provides intermediates to maintain metabolic homeostasis. Deficiency or dysfunction in the mitochondrial electron transport chain causes metabolic disorders (Missaglia *et al*, 2021). Electron transfer flavoprotein (ETF), which is composed of two different subunits, ETFA and ETFB, is the third major electron provider in the mitochondrial electron transport chain after complex I and complex II (Nolfi‐Donegan *et al*, 2020). Mutations in the *ETFA* or *ETFB* genes cause metabolic disorders such as multiple acyl‐CoA dehydrogenase deficiency (MADD) (Yotsumoto *et al*, 2008). ETF functions as a hub that takes up electrons from at least 14 dehydrogenases and feeds them into the mitochondrial respiratory chain. As a result, ETF accepts electrons from dehydrogenases, transfers the electrons to ETF‐ubiquinone oxidoreductase (ETF‐QO) through its cofactors flavin adenine dinucleotide (FAD), and then transports the electrons to the ubiquinone (UQ) pool, and UQ ultimately transfers the electrons to complex III (Nolfi‐Donegan *et al*, 2020). Treatment with riboflavin, a precursor of FAD, could increase ETF stability and thereby partially or fully restore protein function (Zhang *et al*, 2006). However, the mechanism underlying the posttranscriptional regulation of the ETF protein remains unclear.

Here, we found that patient 1 with CAOP syndrome displayed mitochondrial abnormalities. We further discovered that the *S1P* variants severely impair flavination and subsequently destabilize the ETF complex, which consequently disrupts the mitochondrial respiration chain reaction. These mitochondrial abnormalities and the induced phenotypes of CAOP syndrome were significantly improved by supplementation with riboflavin.

## Results

### Clinical features and variants of individuals with CAOP syndrome

**Figure 1:**
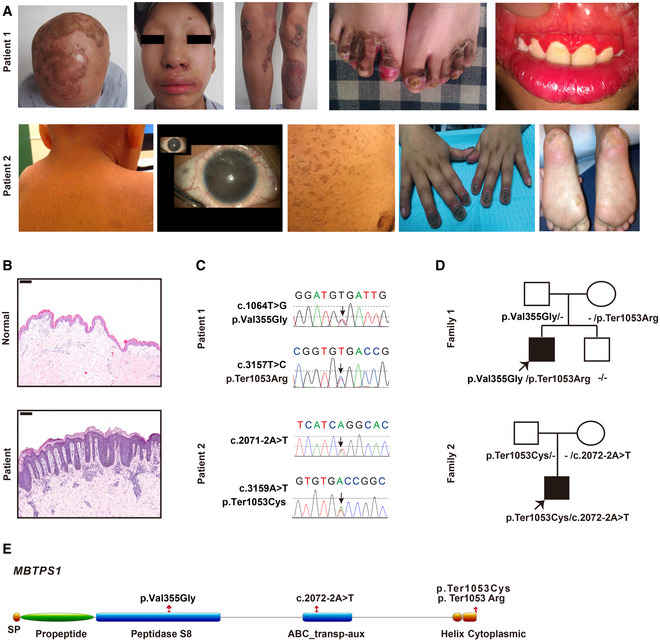
CAOP syndrome patients carry *MBTPS1* variants and inflammatory disorders ARepresentative clinical picture of two patients with CAOP syndrome (14‐year‐old patient 1 and 5‐year‐old patient 2).BHematoxylin and eosin (H&E) staining of skin biopsies from patient 1 and healthy controls. Scale bars: 200 µm.C, DGene sequencing revealed heterozygous *MBTPS1* p.Val355Gly (c.1064T>G) and p.Ter1053Arg (c.3157T>C) variants in patient 1 and heterozygous *MBTPS1* p.Ter1053Cys (c.3159A>T) and c.2072‐2A>T variants in patient 2. The arrows indicate the variants.ESchematic diagram of the S1P domain structure. The p.Val355Gly variant is localized in the peptidase S8 domain, the c.2072‐2A>T variant is located in the ABC transp‐aux domain, and p.Ter1053Arg and p.Ter1053Cys are found in the cytoplasmic domain.

We identified two sporadic cases with CAOP syndrome, a 14‐year‐old Chinese male patient (patient 1) and a 5‐year‐old Hispanic female patient (patient 2) (Table 1). Both patients exhibited mild follicular keratosis, ichthyosis, generalized alopecia, photophobia, red and swollen gums, psoriasis‐like lesions, paronychia, and bilateral cataracts (Fig 1A). Histopathological examination of the disorder also revealed psoriasiform perivasculitis (Fig 1B). Patient 2 showed developmental delay (Appendix Table S1).

**Table 1:** Clinical features in our two patients.

Clinical features	Patient 1	Patient 2	Reference
Skeletal dysplasia	+	−	Kondo *et al* (2018)
Elevated blood lysosomal enzymes	−	ND	Kondo *et al* (2018)
Neurological	−	ND	Kondo *et al* (2018)
Focal myoedema	−	ND	Schweitzer *et al* (2019)
Myalgias	−	ND	Schweitzer *et al* (2019)
hyperCKemia	−	ND	Schweitzer *et al* (2019)
Cutaneous lesions	+	+	Schweitzer *et al* (2019)
Ocular involvement	+	+	NA
Mucosal lesions	+	ND	NA

ND, not detected; NA, not available.

Due to a lower blood zinc level (9.9 µM, normal range: 11–22 µM) combined with alopecia, angular cheilitis, and rash that involved the extremities, perineum, and buttocks, the Chinese patient was previously diagnosed with acrodermatitis enteropathica. He was treated with oral zinc sulfate at 250 mg/day for 6 months at the age of 10 years but failed to respond to oral zinc sulfate, and no *SLC39A4* mutation was detected (Küry *et al*, 2002). We then administered oral acitretin at 20 mg/day for 6 months when patient 1 was 14 years of age with the aim of alleviating psoriasiform lesions, but he again exhibited a poor response. Nonscarring generalized alopecia, psoriasiform lesions, follicular keratosis, ichthyosis vulgaris phenotype, and photophobia can also be found in X‐linked genodermatosis ichthyosis follicularis, atrichia and photophobia (IFAP) syndrome, and keratosis follicularis spinulosa decalvans (KFSD) (Mégarbané & Mégarbané, 2011), and these symptoms have been proven to be caused by mutations in membrane‐embedded zinc metalloprotease/site‐2 protease (*MBTPS2, S2P*) and sterol regulatory element‐binding protein 1 (*SREBP1*). However, no mutations in *MBTPS2* or *SREBP1* were detected in our case.

**Figure EV1:**
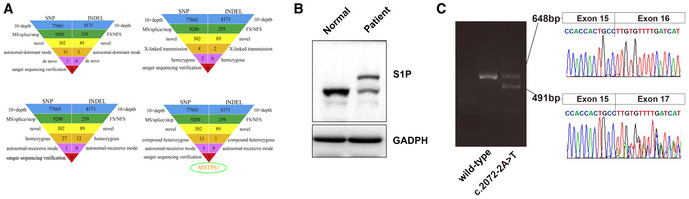
Variants of *MBTPS1* in CAOP syndrome Whole‐exome sequencing analysis of patient 1. No meaningful new variants were detected based on the autosomal dominant pattern, X‐linked hemizygous genetic model, or autosomal recessive inheritance pattern. The analysis according to an autosomal recessive genetic mode detected compound heterozygous variants of the *MBTPS1* gene.Protein expression of S1P in skin biopsies of patient 1 and healthy controls. The heterozygous variants in the *MBTPS1* gene resulted in two bands of S1P protein: one band was similar to the S1P band of the healthy control, and the other band was markedly larger than that of the healthy control.Ethidium bromide‐stained agarose gel of the cDNA products resulting from the splicing assay of the wild‐type (WT) and variant (c.2072‐2A>T) pSPL3*‐MBTPS1* minigenes. The 491‐base‐pair (bp) product represents the mutant RNA transcript lacking *MBTPS1* exon 16, and the 648‐bp band corresponds to a wild‐type transcript that includes *MBTPS1* exon 16. The identity of the PCR fragments was confirmed by sequencing. Source data are available online for this figure.

Whole‐exome sequencing (Fig EV1A) and Sanger sequencing (Fig 1C) identified compound heterozygous variants comprising p. Val355Gly (c.1064T>G) and p. Ter1053Arg (c.3157T>C) in the *MBTPS1* gene (NM_003791.4) in the Chinese patient. The sequencing analyses revealed that both of his biological parents were heterozygous carriers of one *MBTPS1* gene variant and that his healthy brother was negative for both variants (Fig 1D). Similarly, compound heterozygous variants in *MBTPS1* were found in patient 2, and these included p. Ter1053Cys (c.3159A>T) at the same amino acid site as the Chinese patient and c.2072‐2A>T (Fig 1C–E).

We confirmed that the variants identified in patients 1 and 2 were pathogenic by screening a panel of 1200 healthy alleles from public databases and 1,500 additional exomes from internal data. All identified variants were absent from public databases (Data ref: 1,000 genomes, ClinVar, Ensemble, and gnomAD) and healthy controls. The p. Val355Gly (c.1064T>G) variant (patient 1) in the *MBTPS1* gene resulted in a missense mutation of 355 amino acids. The p. Ter1053Arg (c.3157T>C) (patient 1) and p. Ter1053Cys (c.3159A>T) (patient 2) variants in the *MBTPS1* gene led to destruction of the stop codon and production of a protein markedly larger than wild‐type S1P (Fig EV1B). To investigate the role of the c.2072‐2A>T variant (patient 2), we performed a reverse transcription PCR study. Minigene spliced products demonstrated that the presence of the c.2072‐2A>T variant partially abolished the expression of the normal transcript (648‐bp band) and increased the skipping of exon 16 (491‐bp band) (Fig EV1C). Sequencing of these PCR products revealed the presence of two different transcripts.

### S1P deficiency causes abnormal mitochondrial morphology and defective cholesterol metabolism

**Figure 2:**
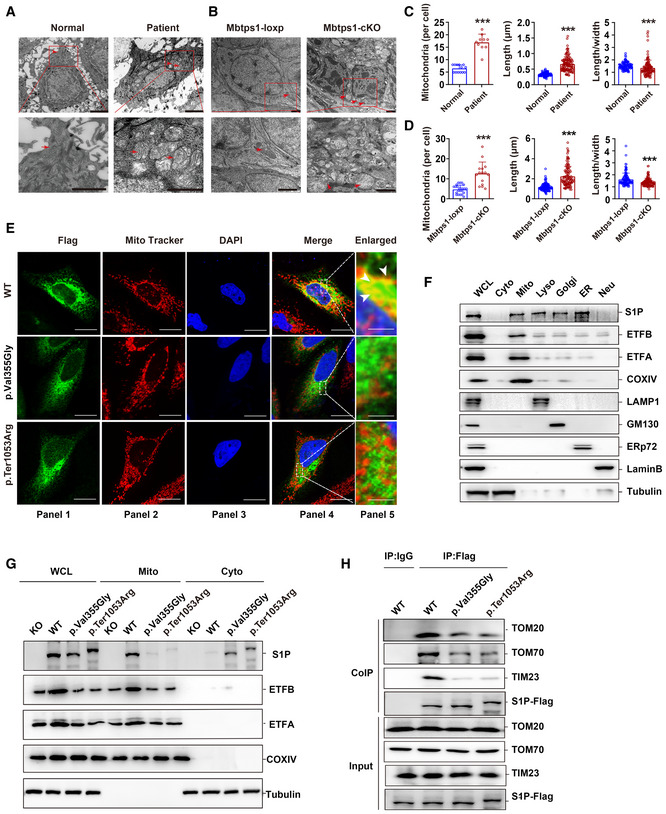
S1P dysfunction impairs mitochondrial import and results in mitochondrial abnormalities A, BRepresentative transmission electron microscopy (TEM) images showing keratinocyte mitochondria (red arrow) in patient 1 (A) and in the *Mbtps1*‐conditional knockout (cKO) mouse model (B). Scale bars: 2 µm.C, DQuantification of the mitochondrial number and morphology in patient 1 (C) and the *Mbtps1*‐cKO mouse model (D). (C) Left panel: *n* = 13 biological replicates of normal individuals, *n* = 10 biological replicates of patient 1. Middle and right panel: *n* = 121 biological samples of normal individuals and patient 1. (D) Left panel: *n* = 23 biological replicates of *Mbtps1‐*loxP mice; *n* = 15 biological replicates of *Mbtps1*‐cKO mice. Middle panel and right panel: *n* = 113 biological replicates of *Mbtps1‐*loxP mice and *Mbtps1*‐cKO.EImmunofluorescence experiments showed that mutant S1P (p.Val355Gly and p.Ter1053Arg) was diffusely localized in the cytosol and showed lower mitochondrial localization compared with wild‐type S1P, which suggested that these variants disrupt its mitochondrial import. The white arrowheads indicate the colocation (yellow) of S1P (green) and the mitochondrial tracer (red). Scale bars: 20 μm (panels 1–4); scale bars: 2 μm (panel 5).FCellular component separation assay showing that S1P was enriched in mitochondria. Biochemical fractionation of the whole‐cell lysate (WCL), cytosol (Cyto, tubulin), mitochondria (Mito, COX IV), lysosomes (Lyso, LAMP1), Golgi apparatus (Golgi, GM130), endoplasmic reticulum (ER, ERp72), and nucleus (Nue, Lamin B) from HaCaT cells.GA component separation assay revealed a lower expression of mutant S1P (p.Val355Gly and p.Ter1053Arg) than wild‐type S1P in mitochondria.HThe impaired binding of mutant S1P (p.Val355Gly and p.Ter1053Arg) to translocase of the outer membrane (TOM) 70 and translocase of the inner membrane (TIM) 23 was detected by a coimmunoprecipitation (Co‐IP) assay. Data information: The data are presented as the means ± SDs. Statistical significance was assessed by the Mann–Whitney two‐tailed *U* test (C and D). ***P < 0.001. Three biological replicates were included in the study (E–H). Source data are available online for this figure.

**Figure EV2:**
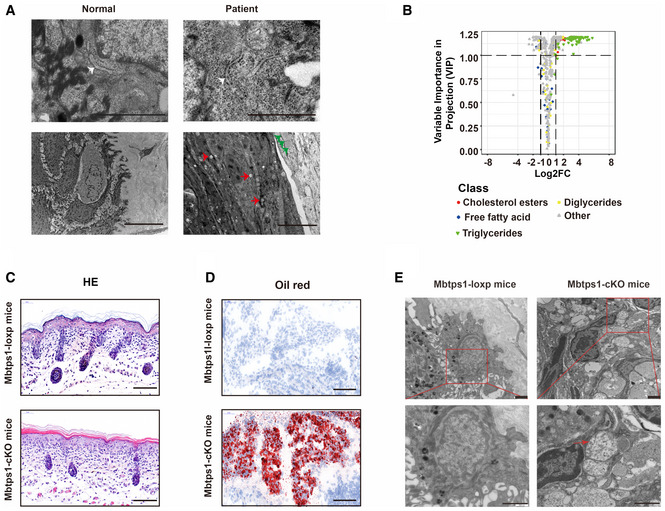
S1P dysfunction results in abnormalities in lipid metabolism and lysosomes Deposited lipid vesicles (green arrowheads, lower panel) and increased amounts of lysosomes (red arrowheads, lower panel) were observed in patient 1, but no ER abnormalities were detected (white arrowheads, upper panel). Scale bars: 1 μm (upper panel) and 5 μm (lower panel).Volcano plot showing abnormalities in cholesterol and triglyceride biosynthesis in *MBTPS1‐*knockout (*MBTPS1*‐KO) HaCaT cells. The fold changes were calculated as log2 (expression in *MBTPS1‐*KO/expression in *MBTPS1‐*Ctrl) (*n* = 4 biological replicates).Hematoxylin and eosin (H&E) staining showed psoriasiform perivasculitis in *Mbtps1‐*conditional knockout (cKO) mice. Scale bars: 100 μm.Oil red O staining showed lipid accumulation in *Mbtps1*‐cKO mice. Scale bar: 100 μm.The encapsulation of mitochondria in lysosomes (red arrowhead) was observed in *Mbtps1‐*cKO mice. Scale bars: 2 μm.

A significant increase in the number of mitochondria (161.5% increase) and morphological abnormalities in the mitochondria (100.7% increase in the length and 10.3% decrease in the length‐to‐width ratio) were observed in the skin lesions of patient 1 by ultrastructural analysis (Fig 2A and C). Moreover, the mitochondrial lamellar cristae structures were disorganized, indicating severe damage to the mitochondria (Fig 2A). However, no obvious ER abnormality was found in the skin lesions of patient 1 (Fig EV2A, upper panel), which indicated that mitochondrial abnormalities are not induced by whole‐cell disease.

Previous studies have shown that S1P is essential for lysosomal biogenesis (Carvalho *et al*, 2020), and abnormal lysosomes were consistently found in patients (Fig EV2A, lower panel). Lipid deposition was observed in the patient’s skin lesions, as evidenced by increased cytoplasmic lipid droplets (Fig EV2A, lower panel). Correspondingly, we also observed a significant increase in cholesterol esters and triglycerides but no significant changes in free fatty acids and diglycerides involved in cholesterol and fatty acid metabolism in *MBTPS1*‐knockout (*MBTPS1‐*KO) HaCaT cells (an immortalized human keratinocyte cell line) (Fig EV2B).

Mitochondrial abnormalities are sometimes associated with severe inflammatory lesions (Chung *et al*, 2020; Xu *et al*, 2020b; Schilf *et al*, 2021). To investigate whether mitochondrial abnormalities are caused by S1P deficiency, we constructed and crossed mice containing a loxP‐flanked *Mbtps1* gene (*Mbtps1* ^loxp/loxp^ mice) with *K14* ^cre^ mice to generate mice specifically lacking *Mbtps1* in the skin (*Mbtps1* conditional knockout (cKO) mice). Similar to the results found for patients, biopsy of the skin of *Mbtps1‐*cKO mice on the first day after birth also revealed psoriasiform perivasculitis (Fig EV2C), lipid accumulation (Fig EV2D), and mitochondrial abnormalities (Figs 2B and EV2E), which included an increase in the mitochondrial number (180.0% increase) and abnormalities in their morphology (88.8% increase in the length and 14.9% decrease in the length‐to‐width ratio) compared with those of *Mbtps1* ^loxp/loxp^ mice (Fig 2D). Furthermore, the encapsulation of mitochondria in lysosomes, an important hallmark of mitophagy, was observed in *Mbtps1‐*cKO mice (Fig EV2E).

**Figure EV3:**
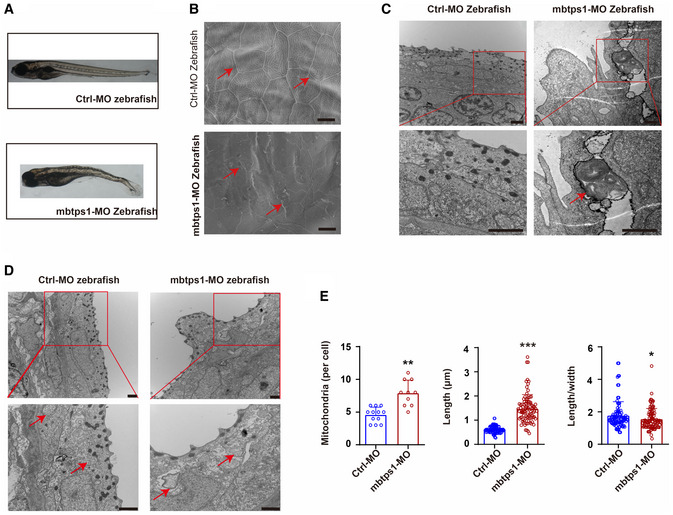
S1P knockdown results in growth limitations, spinal curvature, and skin abnormalities in zebrafish *Mbtps1*‐morpholino (MO) zebrafish exhibited obvious growth limitations and spinal curvature.Skin abnormalities (red arrows) were observed in *mbtps1*‐MO zebrafish. Scale bars: 10 μm.The encapsulation of mitochondria in lysosomes (red arrows) was observed in *mbtps1*‐MO zebrafish at 48 hpf. Scale bars: 2 μm.Increases in the mitochondrial number and morphological alterations of mitochondria were observed in the *mbtps1*‐MO zebrafish model. Mitochondria were induced into multilamellar globules, and the folds of cristae in the inner membrane extended to onion‐like circles in the *mbtps1*‐MO zebrafish model (red arrows). Scale bars: 2 µm.Quantification of the mitochondrial number and morphology in the zebrafish model. Left panel: Quantification of the mitochondrial number per cell in the zebrafish model (*n* = 12 biological replicates of control‐MO (Ctrl‐MO) zebrafish; *n* = 10 biological replicates of *mbtps1*‐MO zebrafish, biological replicates). Middle panel: Quantification of the mitochondrial length in the zebrafish model (*n* = 93 biological replicates). Right panel: Quantification of the length‐to‐width ratio of mitochondria in the zebrafish model (*n* = 93 biological replicates). Data information: The data are presented as the means ± SDs. Statistical significance was assessed by unpaired two‐tailed Student’s *t*‐test (E, left panel) or the Mann–Whitney two‐tailed *U* test (E, middle panel and right panel). **P < 0.01, ***P < 0.001. Source data are available online for this figure.

An *mbtps1*‐knockdown zebrafish model was generated using an antisense morpholino (*mbtps1*‐MO) and showed skeletal deformities, delayed development (Fig EV3A), and skin abnormalities (Fig EV3B). Consistent with the results found for *Mbtps1*‐cKO mice, mitophagy (Fig EV3C) and mitochondrial abnormalities (Fig EV3D and E) were also observed in *mbtps1*‐MO zebrafish.

### S1P is a novel mitochondrial protein whose mitochondrial import is disrupted by *MBTPS1* variants

S1P has been previously reported to be ubiquitously expressed in lysosomes, the Golgi apparatus, and ER (Marschner *et al*, 2011; Kondo *et al*, 2018), but whether S1P exists in mitochondria remains unclear. After showing that S1P dysfunction leads to mitochondrial abnormalities, confocal immunofluorescence indicated that S1P colocalized with the mitochondrial tracer (Fig 2E, upper panel). Furthermore, S1P was enriched in mitochondria, as demonstrated by a cellular component separation assay (Yoo *et al*, 2020) (Fig 2F). We further demonstrated the interaction of S1P with the mitochondrial translocase complex, a general entrance of mitochondrial proteins (Moro *et al*, 1999; Shiota *et al*, 2015) by coimmunoprecipitation (Co‐IP) and found that S1P binds to translocase of the outer membrane (TOM) 20, TOM 70, and translocase of the inner membrane (TIM) 23 (Fig 2H, lane 2). In addition, we tested whether S1P is located in the mitochondrial matrix through a proteinase K (PK) assay of isolated mitochondria (Denuc *et al*, 2016). S1P could still be detected after elimination of the mitochondrial outer membrane, which indicated that S1P resides in the mitochondrial matrix space (Appendix Fig S1).

To evaluate the function of *MBTPS1* variants, we constructed two HaCaT‐cell lines with *MBTPS1* mutations, namely the *MBTPS1* p. Val355Gly and p. Ter1053Arg mutations. We then performed immunofluorescence experiments and found that both S1P (p. Val355Gly) and S1P (p. Ter1053Arg) were diffusely localized in the cytosol and showed less mitochondrial localization compared to wild‐type S1P, which suggested that the *MBTPS1* variants disrupted their mitochondrial import (Fig 2E, middle and lower panels). The component separation assay also showed that S1P variants (p. Val355Gly and p. Ter1053Arg) were expressed at lower levels in the mitochondria and higher levels in the cytosol than wild‐type S1P (Fig 2G). Consistently, the impaired binding of *MBTPS1* variants (p. Val355Gly and p. Ter1053Arg) to TOM 20, TOM 70, and TIM 23 was detected by the Co‐IP assay (Fig 2H, lanes 3 and 4).

### S1P forms a trimeric complex with ETFA and ETFB proteins

**Figure 3:**
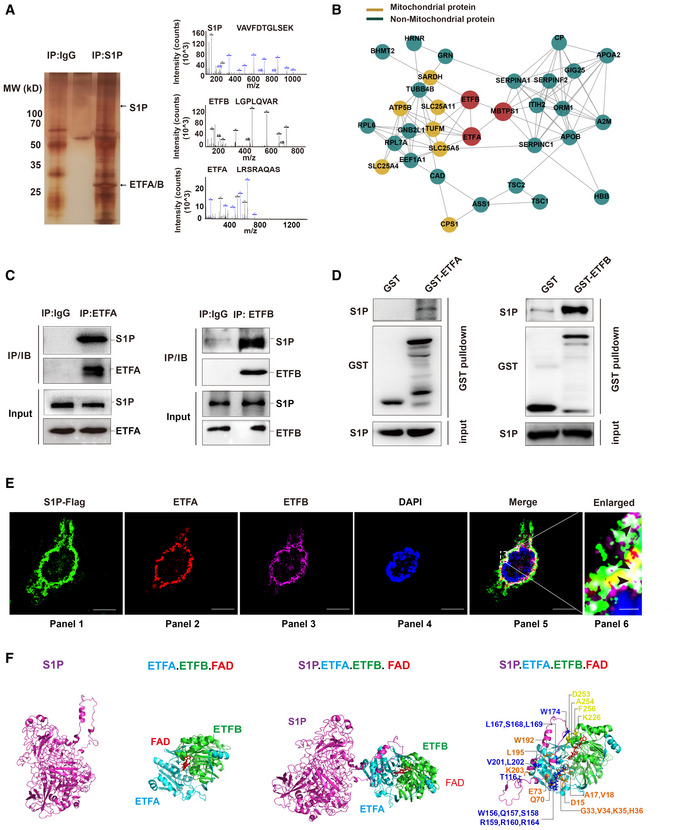
S1P forms a trimetric complex with electron transfer flavoprotein (ETF) A and ETFB proteins LC–MS/MS analysis of S1P‐binding proteins. Total protein lysate was subjected to coimmunoprecipitation (Co‐IP) with normal IgG or S1P antibody. The purified protein complex was separated by SDS–PAGE and then subjected to silver staining. The arrows indicate the bands containing S1P, ETFA, and ETFB. The differential bands were then analyzed by liquid chromatography‐tandem mass spectrometry (LC–MS/MS).Role of the S1P‐ETFA‐ETFB interaction network in mitochondrial and nonmitochondrial protein systems.S1P interacts with endogenous ETFA and ETFB in HaCaT cells. Total protein lysate was subjected to Co‐IP with normal IgG, ETFA (left panel), or ETFB (right panel) antibody. The interaction between S1P and ETFA/ETFB was then detected by immunoblotting.The direct interaction between S1P and ETFA‐ETFB was validated by a GST pull‐down assay. Left panel: *in vitro*‐translated S1P was pulled down by purified GST‐ETFA fusion protein. Right panel: *in vitro*‐translated S1P was pulled down by purified GST‐ETFB fusion protein.Confocal immunofluorescence demonstrated that S1P colocalized with ETFA and ETFB in HaCaT cells. The black arrowheads indicate the colocalization (white) of S1P (green), ETFA (red), and ETFB (purple). Scale bars: 20 μm (panels 1–5); scale bars: 2 μm (panel 6).Three‐dimensional structure of the S1P‐ETFA‐ETFB‐FAD complex *in stereo*. The structures of S1P (magenta), ETFA (light blue), ETFB (green), and FAD (red) are depicted in carbon.

To explore the molecular mechanism underlying the inflammatory symptoms, we screened S1P interactors by Co‐IP and liquid chromatography‐tandem mass spectrometry (LC–MS/MS) and identified 29 potential candidates (Hosp *et al*, 2015) (Fig 3A, Appendix Table S2). Among them, ETFA and ETFB were identified by 6 and 15 unique peptides, respectively. To this end, we performed an in‐depth bioinformatics analysis of potential S1P‐binding proteins using HIPPIE software (Schaefer *et al*, 2012). The protein–protein interaction network suggested that ETF might play a central role in S1P‐associated mitochondrial signaling (Fig 3B). Riboflavin deficiency is known to cause cataracts, photophobia, stomatitis, hyperkeratosis, and delayed development, which are symptoms comparable to those observed in our patients (Baum *et al*, 1942; Barthelemy *et al*, 1986; Balasubramaniam *et al*, 2019; Wortmann *et al*, 2020; Xu *et al*, 2020a). We demonstrated a stable association between S1P and the ETFA/ETFB complex in HaCaT cells through a Co‐IP assay (Fig 3C, Appendix Fig S2A). Correspondingly, IVD, LYRM5, SARDH, and ACADS, known interactors with ETF, were immunoprecipitated from HaCaT cells with anti‐ETFB antibodies (Kim & Miura, 2004; Floyd *et al*, 2016) (Appendix Fig S2B). S1P associated with SARDH (a known ETF substrate) in HaCaT cells (Appendix Fig S2C). The direct interaction between S1P and ETFA/ETFB was then confirmed by the glutathione‐S‐transferase (GST) pull‐down assay (Fig 3D).

Confocal immunofluorescence further demonstrated that S1P colocalized with ETFA/ETFB in HaCaT cells (Fig 3E). A computational 3D complex structural model was generated based on the X‐ray crystal structure from the Protein Data Bank. Docking simulation data suggested that the amino acids TRP‐174 of S1P and LYS‐226, ASP‐253, ALA‐254, and PHE‐256 of ETFA are responsible for the interaction between S1P and ETFA and that the amino acids THR‐116, TRP‐156, GLN‐157, SER‐158, SER‐159, ARG‐160, ARG‐164, LEU‐167, SER‐168, LEU‐169, TRP‐174, VAL‐201, and LEU‐202 of S1P and the amino acids ARG‐12, ASP‐15, TYR‐16, GLY‐33, VAL‐34, LYS‐35, HIS‐36, GLN‐70, GLU‐73, TYR‐192, LEU‐195, and LYS‐203 of ETFB are responsible for the interaction between S1P and ETFB. The “hairpin” structure formed by these amino acids wraps FAD in the complex (Fig 3F). Together, these results imply that S1P forms a trimeric complex with ETFA/ETFB.

### *MBTPS1* dysfunction impairs ETF stability independent of its proteolytic activity

**Figure 4:**
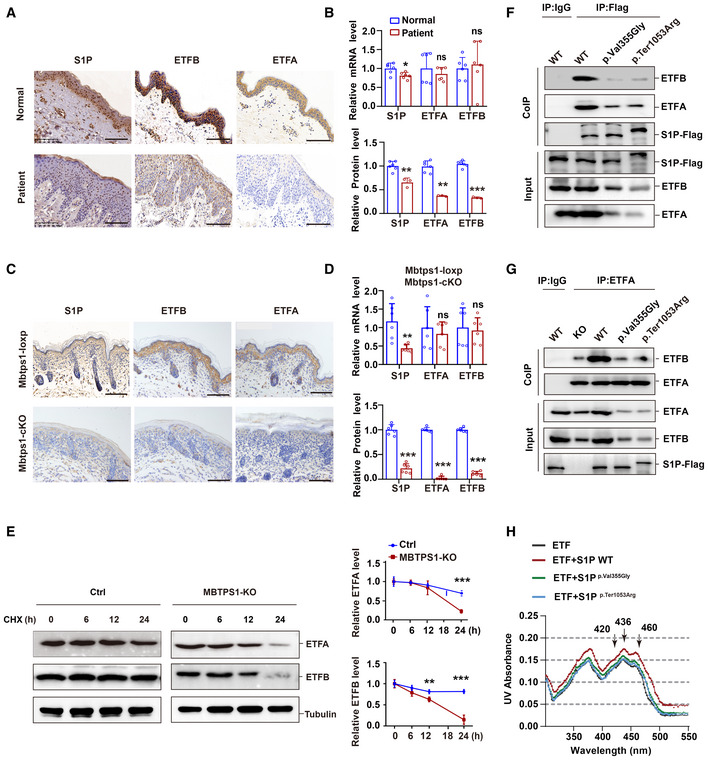
S1P dysfunction impairs ETF flavination and stability Representative immunohistochemical images of S1P, ETFA, and ETFB in normal controls and patient 1. Scale bars: 100 μm.Unchanged mRNA levels and decreased protein levels of ETFA and ETFB were observed in the skin biopsy of patient 1. Quantitative RT–PCR analysis (upper panel, *n* = 6 biological replicates of normal controls; *n* = 6 technical replicates of patient 1) and immunohistochemistry (lower panel, *n* = 6 biological replicates; *n* = 3 technical replicates) of the S1P, ETFA, and ETFB levels.Representative immunohistochemistry images of S1P, ETFA, and ETFB in both *Mbtps1*‐conditional knockout (cKO) and *Mbtps1*‐loxP mice. Scale bars: 100 μm.
*MBTPS1* gene knockout led to decreases in the ETFA and ETFB protein levels *in vivo*. Quantitative RT–PCR analysis (upper panel, *n* = 6 biological replicates) and immunohistochemistry (lower panel, *n* = 6 biological replicates) of the S1P, ETFA, and ETFB levels in *Mbtps1*‐cKO mice and *Mbtps1*‐loxP mice.Cycloheximide (CHX) chase analysis showed that *MBTPS1* knockout induced rapid degradation of ETFA and ETFB proteins in HaCaT cells. Left panel: Representative western blotting images of the ETFA and ETFB protein levels during CHX chase. Right panel: Quantification of the immunoblotting results corresponding to the left panel (*n* = 3 biological replicates).Mutant S1P (p.Val355Gly and p.Ter1053Arg) only weakly interacted with ETFA and ETFB. We constructed Flag‐tagged wild‐type and mutant S1P HaCaT cell lines and then performed a Co‐IP assay with Flag antibody, and the interaction between S1P and ETFA/ETFB was detected by immunoblotting.Wild‐type S1P, but not mutant S1P (p.Val355Gly and p.Ter1053Arg), decreased the association between ETFA and ETFB. We performed a Co‐IP assay with an ETFA antibody, and the interaction between ETFA and ETFB was detected by immunoblotting.Wild‐type S1P, but not mutant S1P (p.Val355Gly and p.Ter1053Arg), enhanced the incorporation of FAD into the ETF complex. The visible spectra of flavin show two shoulder peaks at 420 and 460 nm, indicating the incorporation of FAD in the ETF complex. In the presence of the wild‐type S1P protein, the two peaks were shifted by 0.02 OD units, whereas the additional mutant S1P (p.Val355Gly and p.Ter1053Arg) weakly shifted the two peaks by 0.005–0.01 OD units. Data information: The data are presented as the means ± SDs. Statistical significance was assessed by unpaired two‐tailed Student’s *t*‐test (B: upper panel, B: lower panel for S1P and ETFB, D: upper panel, D: lower panel for S1P and ETFB, E: right panel) or the Mann–Whitney two‐tailed *U* test (B: lower panel for ETFA, D: lower panel for ETFA). ns, not significant, *P < 0.05, **P < 0.01, ***P < 0.001. Three biological replicates were included in the experiment (F, G). Source data are available online for this figure.

**Figure EV4:**
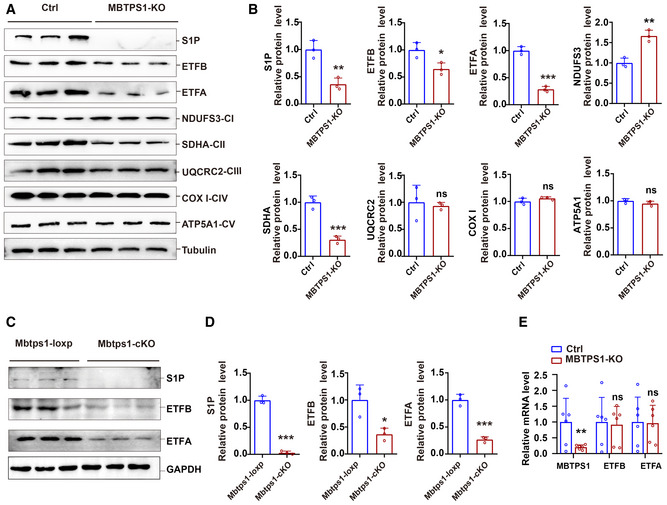
S1P knockout decreases the protein levels of ETF A, BS1P knockout decreased the levels of ETF and the complex II subunit SDHA and increased the protein levels of the complex I subunit NDUFS3 in HaCaT cells. A: Representative immunoblots of S1P, ETFA, ETFB, and mitochondrial electron transport chain proteins in control (Ctrl) and *MBTPS1‐*knockout (KO) HaCaT cells. B: Quantification of the immunoblotting results shown in (A) (*n* = 3 biological replicates).C, DLoss of S1P led to a decrease in ETFA and ETFB proteins *in vivo*. C: Representative immunoblots of S1P, ETFA, and ETFB in *Mbtps1*‐cKO and *Mbtps1*‐loxP mice. D: Quantification of the immunoblotting results shown in (C) (*n* = 3 biological replicates).ELoss of S1P did not affect the mRNA expression of *ETFA* and *ETFB* in HaCaT cells. Quantitative RT–PCR analysis of the S1P, *ETFA,* and *ETFB* mRNA levels in Ctrl and *MBTPS1*‐knockout (KO) HaCaT cells (*n* = 6 biological replicates). Data information: The data are presented as the means ± SDs (B, D, and E). Statistical significance was assessed by unpaired two‐tailed Student’s *t*‐test (B, D, and E). ns, not significant, *P < 0.05, **P < 0.01, ***P < 0.001. Source data are available online for this figure.

Decreased protein levels of ETFA and ETFB were observed in the skin biopsy of patient 1 compared with the control levels (Fig 4A and B), but no significant changes in the mRNA expression of *ETFA* and *ETFB* were detected (Fig 4B). Similar decreases in the protein levels of ETFA and ETFB were observed in *MBTPS1‐*KO HaCaT cells (Fig EV4A and B) and *Mbtps1*‐cKO mice (Figs 4C and D, and EV4C and D). *MBTPS1* knockout did not affect the mRNA level of ETF (Fig EV4E) but induced the rapid degradation of ETF in HaCaT cells, which indicated that S1P may maintain the protein stability of ETF (Fig 4E).

**Figure EV5:**
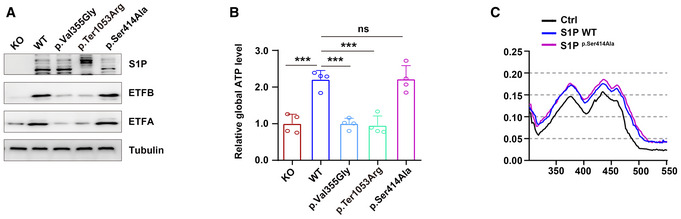
S1P regulates ETF stability independent of its proteolytic activity Both wild‐type and protease‐inactive mutant S1P (p.Ser414Ala) increased the ETFA and ETFB protein levels, indicating that S1P regulates ETF stability independent of its protease activity. Three biological replicates were included in this study.S1P increased global ATP production independent of its protease activity (*n* = 4 biological replicates).S1P enhanced the incorporation of FAD into the ETF complex independent of its protease activity. Data information: The data are presented as the means ± SDs. Statistical significance was assessed by ordinary one‐way ANOVA. ns, not significant, ***P < 0.001. Source data are available online for this figure.

S1P was previously found to act as a key enzyme in cholesterol metabolism and lysosome biogenesis (Marschner *et al*, 2011; Kondo *et al*, 2018; Wang *et al*, 2020). To investigate whether S1P regulates the ETF protein levels via its proteolytic activity, we constructed HaCaT cells expressing wild‐type *MBTPS1*, the protease‐inactive mutant *MBTPS1* (p. Ser414Ala), or *MBTPS1* variants (p. Ter1053Arg or p. Val355Gly) and found that wild‐type *MBTPS1*, but not *MBTPS1* variants (p. Ter1053Arg or p. Val355Gly), increased the protein levels of ETFA and ETFB. Notably, the protease‐inactive mutant *MBTPS1* (p. Ser414Ala) also increased the protein levels of ETFA and ETFB, ATP production, and ETF flavination, which indicated that S1P maintains ETF stability independent of its proteolytic activity (Fig EV5A–C). Notably, proteases can also have protease‐independent functions (Liot *et al*, 2006; Grenell *et al*, 2019). For example, the functions of angiotensin‐converting enzyme 2 (ACE2) can be divided into two categories: protease and protease‐independent functions (Bourgonje *et al*, 2020; Liu *et al*, 2020; Zhou *et al*, 2020). In addition, signal peptide peptidase‐like 3 (SPPL3) functions in innate or adaptive immunity by cleaving proteins involved in antigen presentation. In addition to its proteolytic activity, SPPL3 also interacts with STIM1 and promotes the association of STIM1 with ORAI1 protein through a protease‐independent mechanism (Makowski *et al*, 2015).

### S1P promotes ETFA/ETFB association and subsequent ETF flavination

We observed less binding of mutant S1P (p. Val355Gly and p. Ter1053Arg) to ETFA and ETFB compared with wild‐type S1P, which suggested that *MBTPS1* variants lose the ability to form a trimeric complex with ETF (Fig 4F). The association between ETFA and ETFB reportedly prevents their degradation and maintains their stability via FAD‐mediated flavination (incorporation of the FAD cofactor in ETF after formation of the ETF complex) (Rodrigues & Gomes, 2012). We found that wild‐type S1P, but not the mutant S1P (p. Val355Gly and p. Ter1053Arg), increased the association between ETFA and ETFB, which indicated that S1P strengthened the conformational stability of ETF (Fig 4G). In addition, S1P reduced the association between ETFB and LYRM5 (a deflavinase of ETF) (Floyd *et al*, 2016) but increased the association between ETFB and ACADS (a known ETF substrate) (Kim & Miura, 2004) (Appendix Fig S2D).

The incorporation of FAD in ETF can be seen by the peaks at 420 and 460 nm in the absorbance spectrum (Floyd *et al*, 2016), and we found that wild‐type S1P, but not mutant S1P (p. Val355Gly and p. Ter1053Arg), promoted the incorporation of the FAD cofactor in ETF, as indicated by the significantly enhanced 420 and 460 nm peaks in the presence of wild‐type S1P (Fig 4H). Taken together, these results indicate that wild‐type S1P, but not variant S1P, promotes ETFA/ETFB association and subsequent ETF flavination and thus maintains its stability.

### Loss of S1P impairs mitochondrial respiration and increases the glycolytic capacity

ETF is the third major electron provider in the mitochondrial electron transport chain, after complex I and complex II (Malecki *et al*, 2015). We investigated the effects of S1P on mitochondrial respiration by western blotting and found that S1P knockout decreased the levels of ETF and the complex II subunit SDHA in HaCaT cells, which indicated a tight functional connection between S1P/ETFA/ETFB and complex II. Furthermore, increased levels of the complex I subunit NDUFS3 were also observed, which may indicate a compensatory response of the respiratory chain (Fig EV4A). No significant changes in the complex III subunit UQCRC2, complex IV subunit COX I, or complex V subunit ATP5A1 were observed.

**Figure 5:**
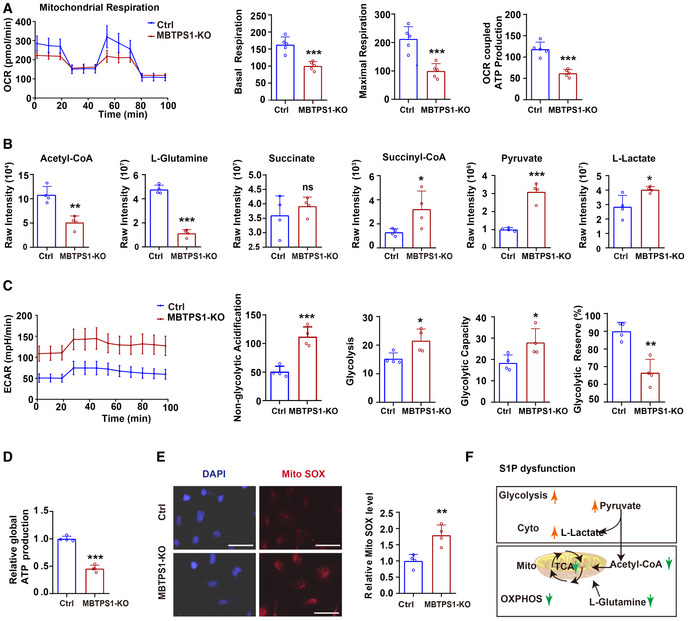
S1P dysfunction impairs cellular OXPHOS and increases glycolysis Mitochondrial respiration (OCR) in control (Ctrl) and *MBTPS1‐*knockout (KO) HaCaT cells were quantified in real time using a Seahorse extracellular flux analyzer. Right subpanels: Quantification of basal respiration, maximal respiration, and OCR‐coupled ATP production in mitochondrial respiration (*n* = 5 biological replicates).Quantification of relevant metabolites in mitochondrial oxidative phosphorylation (OXPHOS) and glycolysis in Ctrl and *MBTPS1‐*KO HaCaT cells (*n* = 4 biological replicates).Nonmitochondrial respiration (ECAR) was quantified in real time using a Seahorse extracellular flux analyzer. Quantification of non‐glycolytic acidification, glycolysis, glycolytic capacity, and glycolytic reserve in Ctrl and *MBTPS1‐*KO HaCaT cells (*n* = 4 biological replicates).
*MBTPS1* knockout led to a decrease in global ATP production in HaCaT cells. Control and *MBTPS1*‐KO HaCaT cells were cultured in six‐well dishes. A standard curve was generated to calculate the sample ATP concentrations using an ATP Lite Luminescence Assay kit (*n* = 4 biological replicates).
*MBTPS1* knockout significantly increased the generation of mitochondrial reactive oxygen species (Mito SOX) in HaCaT cells. Left panel: Representative immunofluorescence images of Mito SOX in HaCaT cells. Scale bars: 100 µm. Right panel: The immunofluorescence intensity was quantified to calculate the relative Mito ROS level in HaCaT cells (*n* = 4 biological replicates).Schematic representation of OXPHOS and glycolysis upon S1P dysfunction. Data information: The data are presented as the means ± SDs. Statistical significance was assessed by unpaired two‐tailed Student’s *t*‐test (A–E). ns, not significant; *P < 0.05; **P < 0.01; ***P < 0.001. Source data are available online for this figure.

Measurement of the oxygen consumption rate (OCR) demonstrated that mitochondrial respiration was diminished in *MBTPS1‐*KO HaCaT cells compared with the control cells, as evidenced by a 38.3% reduction in basal respiration, a 53.1% reduction in the maximal respiratory capacity, and a 47.6% reduction in OCR‐coupled ATP production (Fig 5A). We used targeted steady‐state metabolomics to investigate how S1P influences energy metabolism. Loss of S1P led to a significant reduction in the two carbon sources for the tricarboxylic acid (TCA) cycle (acetyl‐CoA decreased to 47.1%, and l‐glutamine decreased to 23.8%) (Fig 5B, panel 1–2). No significant change in the concentration of succinate was observed (Fig 5B, panel 3), and moderate accumulation of metabolic intermediates in the TCA cycle, such as succinyl‐CoA (by 147.8%) (Fig 5B, panel 4), citrate (by 56.2%), oxaloacetate (by 55.8%), and α‐ketoglutarate (KG) (by 100.1%), was observed in HaCaT cells (Appendix Fig S3A). These findings suggest that loss of S1P impairs mitochondrial respiration and TCA cycle activity.

Furthermore, we observed a compensatory increase in glycolytic metabolites, such as pyruvate (by 205.3%), l‐lactate (by 41.7%) (Fig 5B, panels 5–6), D‐fructose 1,6‐bisphosphate (FDP) (by 50.7%), dihydroxyacetone phosphate (DHAP) (by 2160.4%), and phosphoenolpyruvate (PEP) (by 578.3%) (Appendix Fig S3B), in *MBTPS1‐*KO HaCaT cells compared with the control. We further measured the extracellular acidification rate (ECAR), which mainly represents the glycolytic flux to lactate. We observed an increase in the ECAR in *MBTPS1‐*KO HaCaT cells compared with the control (Fig 5C). In addition, a 54.3% decrease in global ATP production was observed in *MBTPS1‐*KO HaCaT cells compared with control cells (Fig 5D), which indicated an impaired cellular total energy metabolism. Moreover, wild‐type S1P, but not mutant S1P (p. Ter1053Arg or p. Val355Gly), increased global ATP production. Notably, the protease‐inactive mutant S1P (p. Ser414Ala) also increased global ATP production, which indicated that S1P regulates global ATP production independent of its proteolytic activity (Fig EV5B). The destruction of mitochondrial metabolism disrupts intracellular mitochondrial redox homeostasis. Consistently, we observed a 79.2% increase in the generation of mitochondrial superoxide in *MBTPS1‐*KO HaCaT cells (Fig 5E).

In addition to the delivery of electrons to the ubiquinone pool in the mitochondrial respiratory chain, ETF accepts electrons from acyl‐CoA dehydrogenases for fatty acid β‐oxidation (FAO) (Salazar *et al*, 1997). Given that S1P maintains the stability of ETF, we measured palmitoyl‐CoA‐induced enzymatic activity in *MBTPS1*‐KO HaCaT cells and controls. *MBTPS1*‐KO HaCaT cells exhibited impaired FAO activity compared with control cells (Appendix Fig S3C).

Together, these results indicate that loss of S1P initiates a metabolic switch characterized by a reduction in mitochondrial respiration (e.g., TCA cycle activity and FAO activity), an increase in glycolysis, and the deviation of pyruvate from fueling the TCA cycle (with less conversion to acetyl‐CoA) to glycolysis (with more conversion to lactic acid) (outlined in Fig 5F).

### Riboflavin therapy rescues the oxidative phosphorylation (OXPHOS) defect by restoring ETF stability

**Figure 6:**
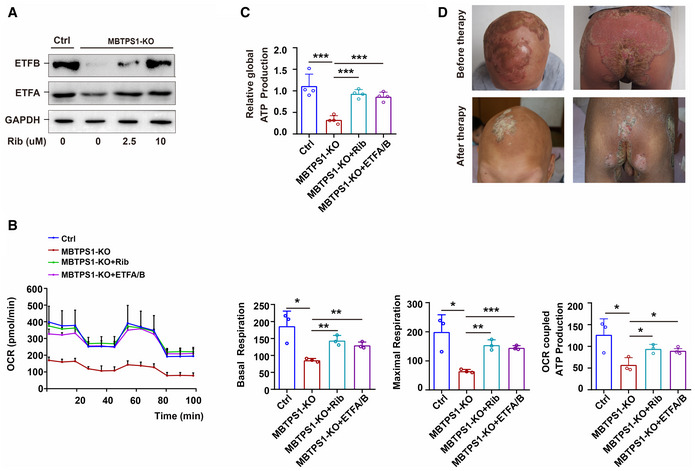
Riboflavin therapy rescues the OXPHOS defect by restoring ETF stability The S1P deficiency‐induced decrease in ETFA and ETFB was significantly reversed by riboflavin (Rib) in a concentration‐dependent manner in *MBTPS1‐*KO HaCaT cells. GAPDH was used as a loading control. *MBTPS1*‐KO cells were initially cultured in DMEM for 24 h and supplemented with 0, 2.5, 5, or 10 µM riboflavin for 3 days. The expression of ETFA and ETFB was then detected by immunoblotting.The S1P deficiency‐induced abnormalities in mitochondrial respiration can be significantly reversed by riboflavin supplementation and ETFA/B overexpression in HaCaT cells. Left panel: Mitochondrial respiration (OCR) was quantified in real time using a Seahorse extracellular flux analyzer. Right panel: Quantification of nonglycolytic acidification, glycolysis, glycolytic capacity, and glycolytic reserve in HaCaT cells (*n* = 3 biological replicates).The decreased global ATP production caused by S1P deficiency could be significantly reversed by riboflavin supplementation and ETFA/B overexpression in HaCaT cells (*n* = 4 biological replicates).Inflammatory lesions in CAOP syndrome were significantly improved by riboflavin supplementation. The representative head image of patient 1 before therapy is shown in Fig 1A (panel 1). Data information: The data are presented as the means ± SDs. Statistical significance was assessed by unpaired two‐tailed Student’s *t*‐test (B, C). *P < 0.05; **P < 0.01; ***P < 0.001. Three biological replicates were included in the experiment (A). Source data are available online for this figure.

FAD acts as a pharmacological chaperone of ETF by improving its conformational stability and biological activity (Henriques *et al*, 2009; Floyd *et al*, 2016). Riboflavin (vitamin B2) is the precursor of FAD. Through flavination and stabilization of ETF, riboflavin therapy has been found to be effective in treating MADD, an autosomal recessively inherited disorder mainly caused by a defect in the *ETF* gene (Gregersen *et al*, 1986; Wen *et al*, 2010; Cornelius *et al*, 2012; Manole *et al*, 2017). Because *MBTPS1* variants impair ETF stability, we propose that riboflavin could be used to reverse the mitochondrial abnormalities induced by *MBTPS1* variants and to treat CAOP syndrome. As expected, the S1P deficiency‐induced decreases in ETFA and ETFB were reversed by riboflavin in a concentration‐dependent manner (Fig 6A), whereas the decreases in mitochondrial respiration and global cell ATP production were restored by riboflavin (Fig 6B and C). Moreover, the S1P deficiency‐induced decreases in mitochondrial respiration and global cell ATP production were also restored by ETFA/ETFB overexpression (Fig 6B and C).

Patient 1 failed to respond to various therapeutic regimens, including 1‐year regular treatment with oral acitretin, supplementation with zinc sulfate, and topical steroids, before the oral administration of riboflavin. Patient 1 was administered oral riboflavin at 3.4–10 mg/day starting when the patient was 14 years of age, and he showed a good response to this treatment. The patient was followed up for 3 years after starting treatment and presented a marked alleviation of severe inflammatory lesions, including oral stomatitis, psoriasis‐like lesions, paronychia, and cheilitis, a slight improvement in photophobia and tongue fissures, and no change in nail dystrophy, alopecia, follicular keratosis, or ichthyosis‐like skin lesions (Fig 6D). These data strongly imply that *MBTPS1* variant‐induced ETF degradation and mitochondrial abnormalities contribute to severe inflammatory lesions in CAOP syndrome.

## Discussion

Previous studies have found that pathogenic variants in *MBTPS2* and *SREBP1* underlie IFAP syndrome by affecting cholesterol homeostasis and epidermal/epithelial cholesterol metabolism (Mégarbané & Mégarbané, 2011; Wang *et al*, 2020). S1P, in cooperation with S2P, participates in cholesterol biosynthesis via cleavage of the sterol regulatory element‐binding proteins (SREBP1 and SREBP2) as well as S1P itself (Yang *et al*, 2001). Here, we found that S1P acts as a mitochondrial protein to regulate the mitochondrial respiratory chain reaction. Our patients with *S1P* variants exhibited severe inflammatory lesions, such as stomatitis, early onset cataract, psoriasiform lesions, and paronychia. These severe inflammatory phenotypes are reportedly associated with mitochondrial dysfunction (Maio *et al*, 2016; Wortmann *et al*, 2020) and were reversed by supplementation with riboflavin in our study.

Collective evidence supports a contribution of mitochondrial abnormalities to the inflammatory lesions noted in CAOP syndrome. First, we identified S1P as a novel mitochondrial protein‐binding partner and stabilizer of the ETFA‐ETFB complex. ETF receives and transfers electrons along the mitochondrial respiratory chain (Malecki *et al*, 2015). Variant S1P loses its ability to flavinylate and stabilize ETF, resulting in impaired electron transfer. Second, the electrons would be free to interact with water or other matrix metabolites and generate reactive oxygen species. Notably, increased mitochondrial reactive oxygen species and a shift from mitochondrial OXPHOS to glycolysis were observed in cells with S1P dysfunction. The metabolic switch plays an important role in the progression of metabolic diseases, although the underlying mechanism is largely unknown (Olsen *et al*, 2007; Hao *et al*, 2009; Maio *et al*, 2016). Our findings suggest that S1P may function as a metabolic switch, which in turn regulates proliferation and environmental adaptation (Gempel *et al*, 2007; Olsen *et al*, 2007; Garone *et al*, 2013; Maio *et al*, 2016). The increases in the mitochondrial size and number of mitochondria observed in this study may be a compensatory response to mitochondrial dysfunction (Floyd *et al*, 2016). Third, riboflavin supplementation has been shown to ameliorate disease progression in patient 1 affected by ETF deficiencies (Cornelius *et al*, 2012), and our results also provide evidence indicating a favorable response of patient 1 with CAOP syndrome to treatment with riboflavin.

More than 90% of all nuclear‐encoded mitochondrial preproteins are sorted into mitochondria by the TOM complex. Our component separation assay and immunofluorescence experiments revealed that S1P was localized in the mitochondria. Coimmunoprecipitation analysis demonstrated that S1P binds to TOM 70, TOM 20, and TIM 23, which indicated that S1P was imported to mitochondria via the TOM and TIM 23 complex (Moro *et al*, 1999; Shiota *et al*, 2015; Thompson *et al*, 2018). Furthermore, our study indicated that the translocation of mutant S1P (i.e., p. Val355Gly and p. Ter1053Arg) to mitochondria via the TOM and TIM 23 complex was decreased. The classic import pathway is triggered by an amphipathic N‐terminal mitochondrial targeting sequence (MTS) recognized by the TOM complex. Precursor proteins with an MTS were further imported by the TIM 23 complex. Unfortunately, no MTS was identified in S1P through NCBI annotation and bioinformatics prediction tools (e.g., Psort2 and MitoProt II). Similarly, previous studies have found that a number of matrix proteins, such as Mrpl32 and Mrp10, do not have a predicted MTS and can be translocated into mitochondria without their MTS (Longen *et al*, 2014; Lehmer *et al*, 2018; Bykov *et al*, 2020).

**Figure 7:**
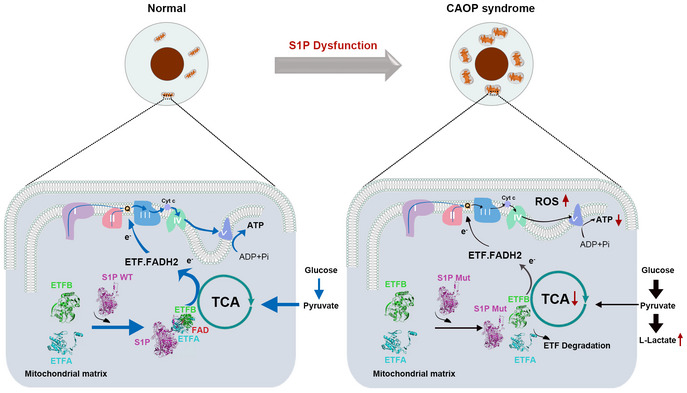
Putative mechanism through which S1P regulates cellular respiration S1P acts as a novel mitochondria‐localized electron transfer flavoprotein (ETF)‐binding partner and is involved in the mitochondrial respiration chain reaction. S1P dysfunction disrupts its translocation to mitochondria, impairs the flavination and stability of ETF, and shifts mitochondrial oxidative phosphorylation (OXPHOS) to glycolysis. OXPHOS, oxidative phosphorylation; ROS, reactive oxygen species. ETF, electron transfer flavoprotein; FAD, flavin adenine dinucleotide; FADH2, reduced flavin adenine dinucleotide; ADP, adenosine diphosphate; ATP, adenosine triphosphate; Q, ubiquinone; TCA cycle, tricarboxylic acid cycle.

Rare cases with neurocognitive delays, three cases with disordered skeletal development, and muscle lesions in another patient have been reported with *MBTPS1* variants (Kondo *et al*, 2018; Schweitzer *et al*, 2019; Carvalho *et al*, 2020; Meyer *et al*, 2020). Our study further indicated that *MBTPS1* variants cause CAOP syndrome. S1P can act as a novel mitochondrial protein‐binding partner and forms a trimeric complex with ETFA and ETFB to enhance the flavination of ETF and maintain its stability (Fig 7). S1P dysfunction‐induced mitochondrial abnormalities may contribute to the severe inflammatory lesions observed in CAOP syndromes.

## Materials and Methods

### Whole‐exome sequencing analysis and pathogenic gene identification

Whole‐exome sequencing analysis and pathogenic gene identification were performed as described previously (Sato‐Matsumura *et al*, 2000; Cambiaghi *et al*, 2002; Thibert *et al*, 2005; Ye, 2011). Comprehensive sequencing data analysis was performed based on the different modes of inheritance in patient 1. The first pattern was autosomal dominant inheritance. Given that the parents of patient 1 were asymptomatic, the proband was assumed to carry a *de novo* pathogenic variant in the disease‐causing gene. Therefore, we filtered out heterozygous variations in his parents. The second pattern was autosomal recessive inheritance. We postulated that the disease‐causing variant was homozygous or compound heterozygous; however, it could also be either a *de novo* variant or a causative variant inherited from the obligate carrier parent. The third pattern was X‐linked inheritance; a hemizygous variant on the X chromosome was assumed to be the genetic defect of the proband. Next, we sequenced the exomes of the two probands via whole‐exome sequencing and performed Sanger sequencing on two probands and their asymptomatic relatives to verify the variants.

This study was approved by Xinhua Hospital, Shanghai Jiaotong University School of Medicine and conducted in the WMA Declaration of Helsinki and the Department of Health and Human Services Belmont Report.

### Antibodies

All antibodies and dilutions used for immunoblotting, immunohistochemistry, immunofluorescence, and immunoprecipitation were described in the Appendix Table S3.

### Generation and culture of cell models

HaCaT cells (Cell Bank of Type Culture Collection Committee of Chinese Academy of Sciences) were seeded in a six‐well dish and transfected with targeted CRISPR/Cas9 plasmids, then reseeded in 96‐well dishes at a density of one cell per well. The knockout efficiency of S1P was verified by immunoblotting. To construct mutant S1P cell lines, *MBTPS1‐*KO HaCaT cells were infected with wild‐type and various mutant *MBTPS1* overexpression viruses. The infected cells were selected in medium with geneticin for 7 days, then harvested and reseeded in 96‐well dishes at a density of one cell per well. The expression efficiency of S1P was detected using a Flag‐tagged antibody.

For the riboflavin supplementation experiment, *MBTPS1‐*KO cells were initially cultured in DMEM for 24 h, and supplemented with 0, 2.5, 5, or 10 µM riboflavin for 3 days before harvesting.

### Mice studies

All animal experiments were performed in accordance with the standards outlined in the National Academy of Sciences Laboratory Animal Care and Use Guidelines. Animal studies were approved by the Animal Studies Committee at Xinhua Hospital Affiliated to Shanghai Jiaotong University School of Medicine. In all experiments, samples (*n*) represent a random selection of a bigger cohort.

Because homozygous germ‐line disruptions of *Mbtps1* in mice were embryonically lethal, we generated mice lacking *Mbtps1* in the skin using the Cre‐loxp recombinase system (Yang *et al*, 2001; Gorski *et al*, 2016). Adult, 7‐ to 10‐week‐old C57BL/6J and *K14‐*Cre mice weighing approximately 20 g (Ellsworth, Jackson Laboratories) were used. *Mbtps1* ^loxp/loxp^ mice were crossed with *K14*‐Cre mice to obtain *Mbtps1* ^loxp/loxp^: *K14*‐Cre (*Mbtps1‐*cKO) mice. Mice were housed under a normal light–dark cycle, and they can eat and drink freely.

### Zebrafish studies

Zebrafish embryos, larvae, and adult fish were raised under standard laboratory conditions at 28.5°C. The wild‐type albino strain of zebrafish was used in this study. To generate *mbtps1* knockdown zebrafish, the *mbtps1*‐MO sequence 5′‐TTGTAGATCCATCTCTCACCTGGCC‐3′ (Gene Tools) was used. Approximately 8.346 ng of *mbtps1*‐MO was microinjected into zebrafish embryos. Control and *mbtps1*‐MO‐treated zebrafish embryos were cultured to 48 h postfertilization (hpf) to evaluate ultrastructural changes in zebrafish skin. Primers for *mbtps1* (forward primer: 5′‐CTGGATGATGGGTTGGGTC‐3′; reverse primer: 5′‐GCTGGCTTGCGGTTTGT‐3′) were used to confirm the knockdown effectiveness.

### Immunoblotting and protein stability

Cell lysates were obtained using RIPA buffer, separated on 8–15% SDS–PAGE gels, and transferred to PVDF membranes (88518; Thermo Fisher Scientific). The bands were blocked in 3% BSA for 1 h, and incubated with the primary antibody overnight. On the second day, the cells were incubated with the secondary antibody for 1 h, and then detected with SuperSignal West Femto Maximum Sensitivity Substrate (34095; Thermo Fisher Scientific).

For protein stability analysis, control (Ctrl) or *MBTPS1‐*KO HaCaT (an immortalized human keratinocyte cell line) (Chen *et al*, 2021) cells were treated with 10 µM cycloheximide dissolved in DMSO for 0, 4, 6, 12, or 24 h.

### Immunofluorescence

HaCaT cells were fixed with 4% paraformaldehyde and permeabilized with 0.3% Triton X‐100. Cells was blocked with 1% BSA for 1h, and then incubated with primary antibodies, secondary antibodies, and DAPI in turn. Fluorescence images were obtained using a confocal microscope (A1, Nikon), and processed with ImageJ (National Institutes of Health), Adobe Photoshop CC (Adobe), and Adobe Illustrator 2019 (Adobe) software.

### LC‐MS/MS analysis

The proteins of interest were enriched using primary antibodies immobilized on Protein G agarose beads. The enriched proteins were separated on 8–12% polyacrylamide gels. Target gel bands were cut out for in‐gel trypsin (T4019; Sigma‐Aldrich) digestion and LC‐MS/MS analysis.

### GST pull‐down assay

GST, GST‐ETFA, and GST‐ETFB fusion proteins were expressed in the bacterial BL21 cells (Sacks *et al*, 2018). GST and GST fusion proteins were purified with Glutathione‐Sepharose 4B beads at 4°C (17‐0756‐01, GE Healthcare Life Sciences). Purified GST fusion proteins were then incubated with endogenously overexpressed Flag‐tagged S1P protein. Finally, the interacting proteins were eluted and detected by western blotting.

### Visible spectra measurements of S1P and ETF

Wild‐type and mutant S1P proteins were expressed in bacterial BL21 cells. The bacterial cells were lysed, and His‐tagged proteins were purified using Ni NTA 6FF beads (20503ES10, Yeasen). Visible spectra of ETF were measured in the presence of wild‐type or mutant S1P (40 mM) in Tris‐HCl buffer (20 mM, pH 8.0).

### Subcellular fractionation

Mitochondria were isolated using the Mitochondria Isolation kit (89874 M, Thermo Fisher Scientific) (Yoo *et al*, 2020). Briefly, 2 × 10^7^ cells were harvested and washed twice with cold PBS. Mitochondria isolation reagents were sequentially added to the samples. The supernatant was then transferred and centrifuged at 4°C. Cytoplasmic protein separation was performed using NE‐PER kit (78835; Pierce). Lysosomes were isolated from cells using the Lysosome Enrichment kit (89839; Pierce). Briefly, 2 × 10^7^ cells were harvested and washed twice with cold PBS. Lysosome isolation reagents were sequentially added to the samples, then the sample was ultracentrifuged for 2 h and sorted out lysosome bands. Golgi was isolated from cells using the discontinuous density gradient separation method with the Golgi Isolation kit (GL0010; Sigma‐Aldrich).

### Quantitative analysis of respiration

Cellular respiration was measured using Seahorse XF24 extracellular flux analyzer (Agilent Seahorse Biosciences). Cells were seeded in DMEM medium and replaced with a pre‐warmed XF assay medium for 1 h. OCR was measured in the Seahorse XF24 analyzer. Basal respiration rate was detected in first block. Next, 1 µM oligomycin, 1 µM carbonyl cyanide 4‐(trifluoromethoxy) phenylhydrazone (FCCP), and 1 µM antimycin were sequentially added to measure block.

For the riboflavin supplementation experiment, *MBTPS1‐*KO cells were initially cultured in DMEM at 37°C with 5% CO_2_ for 24 h and with 5 µM riboflavin for 3 days before harvest.

### Steady‐state metabolomics by LC‐MS/MS

HaCaT cells were extracted for metabolites as previously described with little modification (Grenell *et al*, 2019). Briefly, the extracts were analyzed using Shimadzu LC Nexera X2 UHPLC coupled with a QTRAP 5500 LC MS/MS (AB Sciex). Chromatographic separation was performed with ACQUITY UPLC UPLC BEH Amide analytic column. The mobile phase was performed in buffer A (10 mM ammonium acetate in water, pH 8.8) and buffer B (10 mM ammonium acetate in acetonitrile/water (95/5), pH 8.2). The gradient elution was 95–61% buffer B in 7 min, 61–44% buffer B at 9 min, 61–27% buffer B at 9.2 min, and 27–95% buffer B at 10 min. The column was equilibrated with 95% buffer B at the end. 13C‐nicotinic acid (Toronto Research Chemicals) was added as the internal standard. MultiQuant 3.0.2 software (AB Sciex) was used to integrate the extracted MRM peaks.

### Structural analysis of S1P with ETFA and ETFB

We predicted the single‐chain three‐dimensional structures of wild‐type S1P and ETF (Template PDB ID: 1efv) using the software FR‐t5‐M (Dai *et al*, 2014) and FALCON. S1P and ETFA‐ETFB three‐dimensional structures were docked by ZDock software (Pierce *et al*, 2014). S1P‐ETFA‐ETFB complex model was selected from the 10 candidate complexes using the ZDock score and other computational structure biology methods MEFTop (Dai *et al*, 2014) and QIPI (Dai *et al*, 2016), respectively.

### ATP and MitoSOX measurement

Intracellular ATP was measured using an ATP Lite Luminescence Assay kit (A22066; PerkinElmer). Control and *MBTPS1‐*KO HaCaT cells were cultured in six‐well dishes. A standard curve was generated to calculate the sample ATP concentrations. Intracellular ATP was normalized and presented as a percentage of the control content. Mitochondrial ROS levels were measured by monitoring the intracellular oxidation of the cell‐permeant probe MitoSOX (M36008; Thermo Fisher Scientific). Control and *MBTPS1‐*KO HaCaT cells were cultured in six‐well dishes, and 5 μM MitoSOX reagent was added and incubated for 10 min at 37°C in the dark.

### FAO activity assay

FAO activity was measured using FAO assay kit (E‐141L, Biomedical Research Service Center) according to the manufacturer's instructions.

### Statistical analysis

For *in vitro* and *in vivo* experiments, sample sizes were chosen according to the standard practice in the field. For humans, sample size was limited by the patient’s sample available. In all aminal experiments, samples (*n*) represent a random and blind selection of a bigger cohort. The status of the mice and zebrafish was always known to the experimenters. Statistical analyses were performed using GraphPad Prism software (version 6.0, La Jolla). Results were presented as mean ± SD. Shapiro–Wilk normality test was performed to check the data distribution. *F*‐test was performed to compare data variances. According to the distribution and variances of the data, unpaired two‐tailed *t*‐test or Mann–Whitney two‐tailed test was used for comparison between two groups, and ordinary one‐way ANOVA with Dunnett’s *post‐hoc* or Kruskal–Wallis test followed by Dunn’s *post‐hoc* was used for comparison among multiple groups. A *P* < 0.05 (two‐sided) was considered statistically.

## Author contributions

**Fuying Chen:** Conceptualization; Resources; Data curation; Software; Formal analysis; Supervision; Validation; Investigation; Visualization; Methodology; Writing—original draft; Project administration; Writing—review & editing. **Cheng Ni:** Data curation; Formal analysis; Writing—original draft; Writing—review & editing. **Xiaoxiao Wang:** Conceptualization; Resources; Data curation; Software; Formal analysis; Supervision; Validation; Investigation; Visualization; Methodology; Writing—original draft; Project administration; Writing—review & editing. **Ruhong Cheng:** Resources; Supervision; Investigation; Visualization. **Chaolan Pan:** Methodology; Writing—review & editing. **Yumeng Wang:** Visualization; Writing—review & editing. **Jianying Liang:** Formal analysis; Writing—review & editing. **Jia Zhang:** Visualization; Writing—review & editing. **Jinke Cheng:** Investigation; Writing—review & editing. **Y Eugene Chin:** Resources; Writing—review & editing. **Yi Zhou:** Writing—review & editing. **Zhen Wang:** Methodology; Project administration; Writing—review & editing. **Yiran Guo:** Writing—review & editing. **She Chen:** Methodology; Writing—review & editing. **Stephanie Htun:** Resources; Writing—review & editing. **Erin F Mathes:** Resources; Writing—review & editing. **Alejandra G de Alba Campomanes:** Resources; Writing—review & editing. **Anne M Slavotinek:** Resources; Writing—review & editing. **Si Zhang:** Conceptualization; Resources; Data curation; Software; Formal analysis; Supervision; Validation; Investigation; Visualization; Methodology; Writing—original draft; Project administration; Writing—review & editing. **Ming Li:** Conceptualization; Resources; Data curation; Software; Formal analysis; Supervision; Funding acquisition; Validation; Investigation; Visualization; Methodology; Writing—original draft; Project administration; Writing—review & editing. **Zhirong Yao:** Conceptualization; Supervision; Funding acquisition; Validation; Visualization; Writing—review & editing.

In addition to the CRediT author contributions listed above, the contributions in detail are:

FC and XW were responsible for the study design, data analysis, and drafting of the manuscript. CN, RC, JL, JZ, and YG analyzed the data and wrote the manuscript. XW, YW, and CP were responsible for organelle purification, immunostaining, and mouse model construction. EFM, AGC, SH, and AMS were responsible for the clinical and genetic analysis of patient 2. JC, YEC, SC, YZ, and ZW were responsible for the analysis. SZ, ML, and ZY were responsible for the study concept and design, study supervision, and funding. All authors reviewed manuscript.

## Disclosure and competing interests statement

The authors declare that they have no conflict of interest.

## For more information

The URLs for data presented in this article are as follows:

- 1,000 genomes, http://www.1000genomes.org
- Clinvar, https://www.ncbi.nlm.nih.gov/clinvar
- dbSNP, http://www.ncbi.nlm.nih.gov/projects/SNP
- Ensembl, http://www.ensembl.org
- gnomAD database, https://gnomad.broadinstitute.org
- Human genome build (GRCh37/hg19), https://www.ncbi.nlm.nih.gov/grc
- Human Splice Finder, http://www.umd.be/HSF3
- Online Mendelian Inheritance in Man (OMIM), http://www.omim.org
- PolyPhen‐2, http://genetics.bwh.harvard.edu/pph2
- Primer 3, http://frodo.wi.mit.edu/primer3
- PRoteomics IDEntifications database (PRIDE), https://www.ebi.ac.uk/pride
- RefSeq, https://www.ncbi.nlm.nih.gov/refseq (xix) SIFT, http://sift.jcvi.org
- RCSB PDB, https://www.rcsb.org/

## Supporting information



Appendix

Expanded View Figures PDF

Source Data for Expanded View and Appendix

Source Data for Figure 2

Source Data for Figure 4

Source Data for Figure 5

Source Data for Figure 6

## Data Availability

The here published material and data will be made available in accordance with the relevant ethical standards and legal guidelines. The data sets produced in this study are available in the following databases: Protein interaction AP‐MS data: Protein identifications database (PRIDE) PXD031064 (https://www.ebi.ac.uk/pride/archive/projects/PXD031064). Human variations: ClinVar SCV002050318 (https://www.ncbi.nlm.nih.gov/clinvar/SCV002050318) and ClinVar SCV002050317 (https://www.ncbi.nlm.nih.gov/clinvar/SCV002050317).
